# Influence of Metal Diboride and Dy_2_O_3_ Additions on Microstructure and Properties of MgB_2_ Fabricated at High Temperatures and under Pressure

**DOI:** 10.1038/srep29306

**Published:** 2016-07-13

**Authors:** Y. Yang, M. D. Sumption, E. W. Collings

**Affiliations:** 1 Center for Superconducting and Magnetic Materials, Department of Materials Science and Engineering, the Ohio State University, Columbus, OH, USA

## Abstract

High temperatures and under pressure (HTP) processing has been used to study the effects of chemical doping in MgB_2._ ZrB_2_, TiB_2_ and NbB_2_ were selected as additives since, like MgB_2_, they have an AlB_2_-type structure and similar lattice parameters. Dy_2_O_3_ was selected as it has been reported to generate nanoscale, secondary intragrain phases in MgB_2_. While C is known to enter the B-sublattice readily, attempts to dope Zr and other elements onto the Mg site have been less successful due to slow bulk diffusion, low solubility in MgB_2_, or both. We have used high-temperature, solid-state sintering (1500 °C), as well as excursions through the peritectic temperature (up to 1700 °C), to investigate both of these limitations. Bulk MgB_2_ samples doped with MB_2_ (M = Zr, Ti and Nb) and Dy_2_O_3_ additions were synthesized and then characterized. Lattice distortion and high densities of crystal defects were observed in the MgB_2_ grains around nano-sized MB_2_ inclusions, this highly defected band contributed to a large increase in *B*_*c2*_ but was not large enough to increase the irreversibility field. In contrast, distributed intragrain precipitates were formed by Dy_2_O_3_ additions which did not change the lattice parameters, *T*_*c*_, *T*_*c*_ distribution or *B*_*c2*_ of MgB_2_, but modified the flux pinning.

Extensive efforts have been expended in doping MgB_2_ to enhance its superconductive properties, particularly its upper critical field, *B*_*c2*_. The substitution of C for B has been shown to significantly increase MgB_2_’s *B*_*c2*_ beyond that of the unalloyed sample^1^,^2^,^3^,^4^,^5^,^6^. Unfortunately, C doping is successful only at low temperatures (<20 K) since it reduces *T*_*c*_ and increases electron impurity scattering only in the σ band, leaving the high temperature (>20 K) *B*_*c2*_ unchanged or even reduced. In order to enhance the *B*_*c2*_ of MgB_2_ in the higher temperature regime, many attempts have been made to find effective dopants for Mg-site substitution to increase electron impurity scattering in both the σ band and the π band^7^,^8^, but without much success in terms of improved properties. Although the substitution of Al for Mg has been demonstrated, it was found to reduce *B*_*c2*_ 9,10.

Further studies focusing on the AlB_2_-like metal diborides ZrB_2_, TiB_2_ and NbB_2_, (e.g. refs 11, 12, 13, 14, 15, 16) yielded contradictory results. Feng *et al*.^11^,^12^ reported an enhancement in *B*_*c2*_ in response to 10 mol% Zr doping; Bhatia *et al*.^13^ observed a significant increase in *B*_*c2*_ (from 20.5 T to 28.6 T at 4.2 K) after adding 7.5 mol% ZrB_2_ to MgB_2_ bulks. On the other hand, Zhang *et al*.^14^ reported no *B*_*c2*_ enhancement in ZrB_2_ doped MgB_2_ tapes. In any case, while *B*_*c2*_ enhancements have been noted by various researchers working with MgB_2_ PIT or powder type processes, no one has reported enhanced transport current, suggesting that the effect may be in a surface layer. The one effort to date which has clearly injected Zr deeply into the grain, resulting in a pulsed laser deposition (PLD) synthesized ZrB_2_-doped MgB_2_ thin film^15^,^16^, showed a much stronger response to the presence of Zr, and in this case a decrease of *T*_*c*_ and *B*_*c2*_ with increasing Zr content. These various observations give rise to the question: what is the actual influence of Zr doping in MgB_2_? The possible roles of Zr in MgB_2_ can be summarized in terms of: 1) extrinsic effects, such as modified intergranular connectivity and reduced grain size^11^,^12^; 2) intrinsic effects, such as an influence on *B*_*c2*_ of Zr substitution for Mg^13^,^14^,^15^,^16^, or increased flux pinning by a distribution of nano-sized ZrB_2_/Zr precipitates^14^. On the other hand, incomplete microscopic evidence of Zr substitution for Mg has been provided, at least for materials made by equilibrium processes (contrasting to the non-equilibrium processing of the films of^15^,^16^). Therefore further study on the limits of Zr doping was deemed necessary.

There are many roadblocks to clarifying the true role of chemical doping in MgB_2_. Chief among them is that homogeneous doping is very hard to achieve. Traditional powder synthesis is generally performed at 600–1000 °C –too low to form homogeneously doped samples. To overcome this problem a high temperature under pressure (HTP) route (see below) was developed to explore solubility limits of dopant species in MgB_2_ and maximize diffusion during reaction. MgB_2_ bulks synthesized by HTP should have a greater depth of dopant penetration into the MgB_2_ for any species introduced (if it is soluble) given the increase in diffusion rate at higher temperatures. HTP samples also have large grain size (over 5 μm) making chemical analysis easier. The purpose of the HTP method is to minimize any diffusion limitations so that we can explore the solubility limits of doping, rather than to fabricate MgB_2_ bulk samples with high *J*_*c*_. Also, the use of existing metal diborides (MB_2_) with a structure isomorphous to MgB_2_ (P6/mmm) as a vector for effective metal element doping is a promising way to investigate possible changes of superconducting properties like *B*_*c2*_ and *T*_*c*_. Thus, three sets of MgB_2_ bulks doped with ZrB_2_, TiB_2_ and NbB_2_ powders were prepared.

Most additions which have been attempted for MgB_2_ tend to accumulate at the grain boundaries, with the exception of the above-mentioned C-bearing additions. On the other hand, several studies have shown that a very small amount of Dy_2_O_3_^17^ can form nanosize precipitates within the MgB_2_ grains and thereby enhance flux pinning without changing *T*_*c*_. Thus a Dy_2_O_3_ doped MgB_2_ bulk was also included in this study for comparison. The changes in microstructure and lattice parameter, as well as the superconducting properties *B*_*c2*_, *T*_*c*_ and flux pinning were studied for all samples and are discussed below.

## Results and Discussion

### Influence of MB_2_ and Dy_2_O_3_ doping on XRD and lattice constants

**Figure 1:**
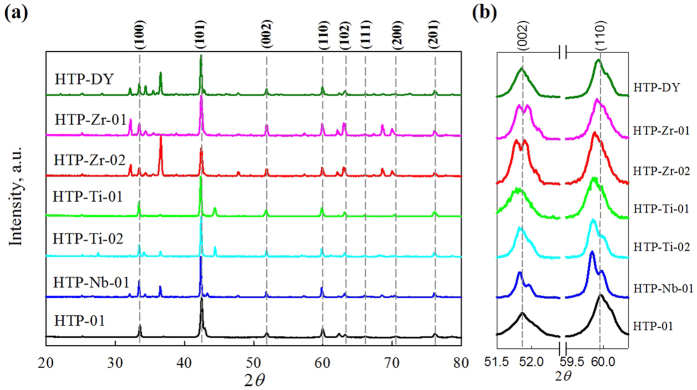
(**a**) X-ray diffraction characterization of undoped MgB_2_ sample HTP-01, and all theMB_2_ doped samples; (**b**) Peaks (110) and (002), as these two peaks are directly related to lattice parameter *a* and *c*, respectively. Note for the MB_2_ doped samples, small peak shifting and MB_2_ peaks are observed, while no peak shifting is observed in the Dy_2_O_3_ doped sample.

The X-ray diffraction data for all HTP bulks are presented in Fig. 1 where the Bragg reflections are indexed for only the MgB_2_ phase for simplicity. MgO and Mg were present at some level in all samples (MgO was less than about 2 wt% for samples reacted below the peritectic). MgB_4_ peaks are also present for samples HT at 1700 °C. Only a very small peak shift (less than 0.2 degree) at both (110) and (002) was observed in MB_2_ doped samples while no peak shift was observed for the Dy_2_O_3_ doped sample. Peaks corresponding to MB_x_ impurity phases were observed in all MB_2_ doped samples. The lattice parameters extracted from pseudo-Voigt fitting the MgB_2_ peak reflections and the calculated lattice parameters by Vegard’s law are given in Table 1. For the Dy_2_O_3_ added sample HTP-DY, similar to Chen’s report^17^, the lattice parameters *a* and *c* did not change with Dy_2_O_3_ addition. Similarly, for the MB_2_ added samples, even though both lattice parameters *a* and *c* were slightly changed, these changes were very small and none of the doped samples obeyed Vegard’s law, unlike C-doped MgB_2_ HTP bulks^6^. Therefore it seems that even under HTP processing the metal borides (ZrB_2_, TiB_2_ and NbB_2_) mainly acted as impurity phases and did not form homogeneous solid solutions with MgB_2_, at least not to an extent detectable by XRD. However, the small changes in lattice parameters suggest that a distortion of the MgB_2_ lattice was present. Such distortion, caused by strain generated around these dopant impurities (see below) rather than elemental substitution, appears to be the driver for the modified upper critical field *B*_*c2*_ of the doped samples.

### Influence of MB_2_ and Dy_2_O_3_ doping on microstructure - SEM and TEM

**Figure 2:**
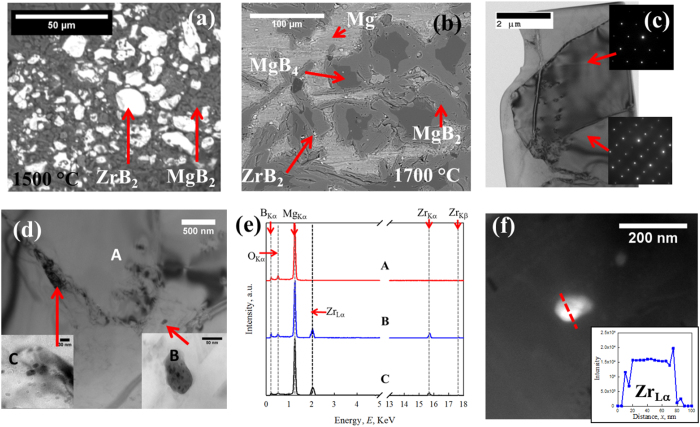
(**a**) BSE image of HTP-Zr-01; (**b**) BSE image of HTP-Zr-02; (**c**) BF TEM image of HTP-Zr-02 (Microstructures of the undoped bulk can be found in^6^,^18^,^19^). Insets are SAD of two distinct MgB_2_ grains; (**d**) nano-size inclusions observed close to/at MgB_2_ grain boundaries; (**e**) EDS spectra of spot A, B and C; (**f**) HAADF image of a nano-size inclusion close to MgB_2_ grain boundaries. Inset is the intensity of Zr_Lα_ from STEM-EDS scanning across the inclusion (red dash line).

**Figure 3:**
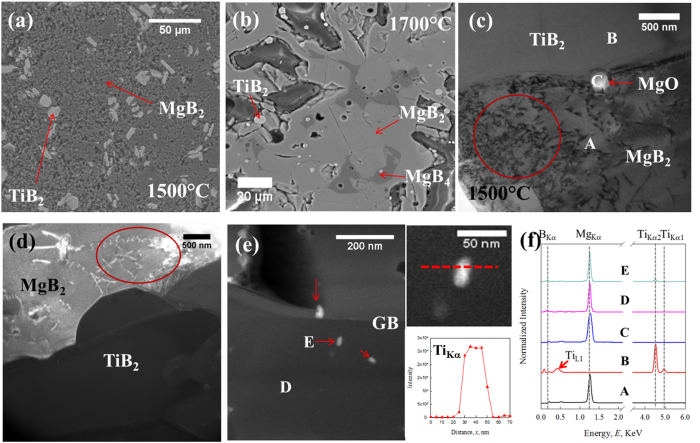
(**a**) BSE image of HTP-Ti-01; (**b**) BSE image of HTP-Ti-02; (**c**) BF TEM image of HTP-Ti-01. Large amount of crystal defects in MgB_2_ grain close to MgB_2_/TiB_2_ interface; (**d**) DF TEM image of ingrain crystal defects in MgB_2_ grain close to TiB_2_; (**e**) HAADF image of MgB_2_ grains with high defect density and STEM-EDS scanning across one of the nano-inclusions (red dash line). Inset is the intensity of Ti_Kα_; (**f**) EDS spectra of spot A-E from (**c,e**).

The results of back-scatter (BSE) SEM characterization performed on the ZrB_2_ doped samples are presented in Fig. 2. The microstructures of HTP-Zr-01 (1500 °C, below the peritectic temperature) and HTP-Zr-02 (1700 °C, above the peritectic temperature) are shown in Figs 2(a) and 3(b) (for comparison, the microstructures of the undoped bulk can be found in^6^,^18^,^19^). Two phases are visible: MgB_2_ (majority phase, dark grey) and ZrB_2_ (minority phase, white) in HTP-Zr-01; while in HTP-Zr-02, Mg and MgB_4_ phases were present since it experienced (upon cooling) the reaction 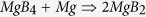.

Figure 2(c) represents the bright-field (BF) TEM image obtained from a thin foil extracted from HTP-Zr-02 (1700 °C). This foil contains a cross section of several grains. The results from the energy dispersive spectroscopy analysis (EDS) and selected area diffraction (SAD) confirm that these large grains are MgB_2_. An intragranular crack and dislocation loops are present in one of the grains. Since HTP-Zr-02 was processed above the peritectic, the crack presumably resulted from volume expansion taking place during cooling as the MgB_4_ converted into MgB_2_. TEM examination revealed a number of impurity phases in the form of 30–80 nm inclusions around the MgB_2_ grains, Fig. 2(d). The results of EDS analysis performed on these inclusions are presented in Fig. 2(e). It is clear that these inclusions contain Zr, or possibly ZrB_2_, which is likely dispersed around the MgB_2_ grains in the bulk. Compared to other areas inside the MgB_2_ grains, the regions around these ZrB_2_ inclusions have much higher contrast under BF condition, which suggests that strain fields were generated around these inclusions and that the MgB_2_ lattice was distorted locally. This local lattice distortion may be the origin of the slight lattice parameter changes observed by XRD analysis in Section 3.1. Figure 2(f) shows HAADF imaging for one of these nano-size inclusions. Since HAADF imaging is sensitive to variations in the atomic number (Z-contrast), these white inclusions should have a higher average atomic number than MgB_2_. A STEM-EDS line scan applied using 21 distinct points over ~100 nm across this inclusion confirmed it was ZrB_2_. The Zr signal dropped to zero quickly outside of the inclusion, beyond the ZrB_2_/MgB_2_ interface. The spatial resolution of the STEM-EDS line scans is about 5 nm, thus these observation indicate that Zr did not notably penetrate into the MgB_2_ lattice.

**Figure 4:**
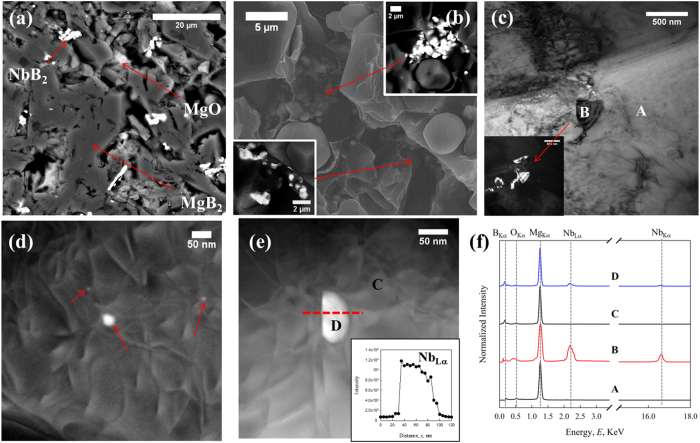
(**a**) BSE image of HTP-Nb-01; (**b**) fractured SE images, insets are BSE image of the fractured area; (**c**) BF TEM image of HTP-Nb-01 contained NbB_2_ inclusions. Inset is the DF TEM image of NbB_2_ inclusions; (**d**) HAADF image of MgB_2_ grains with high density of defects; (**e**) STEM-EDS scanning across one of the nano-inclusions (red dash line). Inset is the intensity of Nb_Lα_ ; (**f**) EDS spectra of spot A-D.

Figure 3 shows the BSE images of the TiB_2_ doped samples, HTP-Ti-01 (1500 °C, below the peritectic) and HTP-Ti-02 (1700 °C, above the peritectic). Similar to the behavior of ZrB_2_, TiB_2_ mainly acts as an impurity phase (light grey in Fig. 4(a,b)) and is widely distributed. A TEM thin foil containing a cross section of both TiB_2_ and MgB_2_ grains was carefully extracted from HTP-Ti-01. A BF image including MgB_2_, TiB_2_, and their interface is represented in Fig. 3(c). A large number of defects can be observed inside the MgB_2_ grains close to the MgB_2_/TiB_2_ interface. Inclusions 100–200 nm in size are found at the interface as well as at MgB_2_ grain boundaries. EDS analysis confirms that these inclusions are MgO, Fig. 3(f). Dark-field (DF) imaging was also used to examine the dislocations and the interface since crystal defects have stronger contrast under DF conditions. In Fig. 3(d), the DF image clearly confirms that the MgB_2_ grain contains a high density of defects. Detailed in-grain analysis was performed using HAADF imaging. Figure 3(e) shows a HAADF image of MgB_2_ grains with high defect density. Nano-size inclusions (~10–30 nm, white) dispersed both in and around MgB_2_ grains were observed. EDS analysis performed on randomly selected white inclusions confirmed that they were TiB_2_ (EDS spectrum of spot E (inclusion) in Fig. 3(f)). No Ti was detected by EDS in the other regions of MgB_2_ grains (EDS spectrum of spot D (matrix) in Fig. 3(f)). STEM-EDS analysis was applied across inclusion E, and beyond the TiB_2_/MgB_2_ interface the intensity of the Ti signal quickly dropped to zero, indicating that Ti did not dissolve into the MgB_2_ lattice. These nano-size TiB_2_ inclusions can also contribute to the high defect density observed in MgB_2_ grains in Fig. 3(c,d).

**Figure 5:**
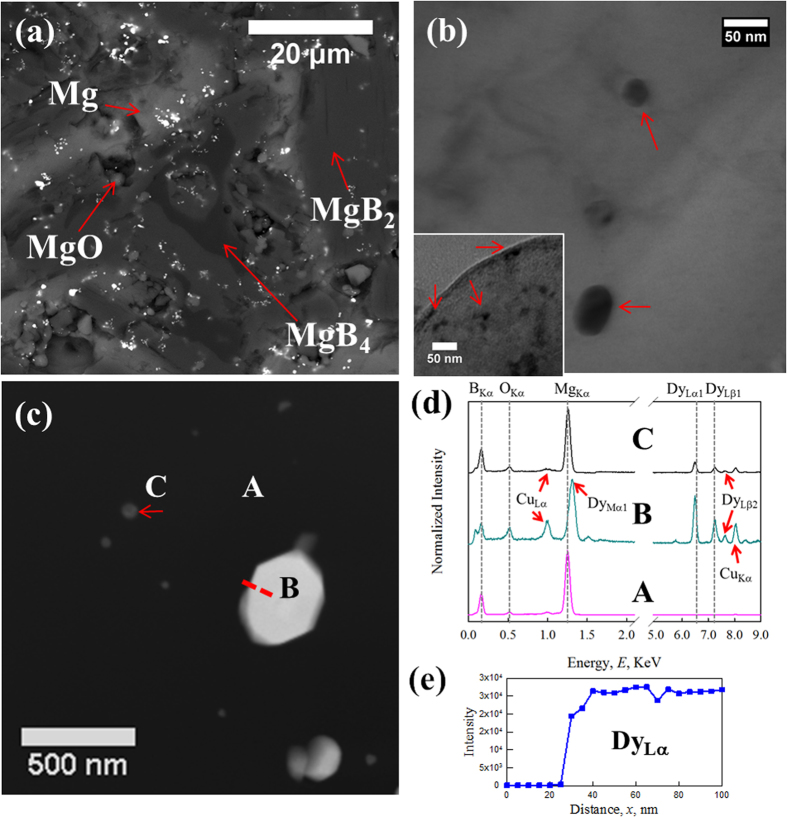
(**a**) BSE image of HTP-DY, four different phases (MgB_2_, MgB_4_, Mg and MgO) are labeled; (**b**) BF TEM image of nano-size inclusions (10–50 nm) are found inside MgB_2_ grains; (**c**) HAADF image of one MgB_2_ grain, white precipitates are Dy-contained; (**d**) EDS spectra of spot A-C in (**c**); (**e**) the intensity of Dy_Lα_ from STEM-EDS scanning (red dash line in (**c**)).

In the NbB_2_-added sample HTP-Nb-01 (HT below the peritectic), three phases are visible in the BSE images of Figs 4(a) and 5(b): MgB_2_ (majority phase, dark grey), MgO (minority phase, light grey) and NbB_2_ (minority phase, white). In Fig. 4(b), based on fractured secondary electron (SE) imaging by ‘through the lens’ (TTL) detection, NbB_2_ particles are observed outside the MgB_2_ grains. These particles are small (~300–500 nm), well connected with the MgB_2_ grains, and dispersed throughout the bulks.

Further analysis was performed on a TEM thin foil sectioned from HTP-Nb-01. Both BF (Fig. 4(c)) and DF images (Inset of Fig. 4(c)) show nano-size inclusions (~300 nm) embedded in the MgB_2_ grain boundaries. Moreover, a large number of defects can be observed inside the MgB_2_ grains around these inclusions, while the other MgB_2_ grains have fewer intragranular defects. The EDS results in Fig. 4(e) confirm that these inclusions are NbB_2_. HAADF imaging performed on MgB_2_ grains with high density of defects is presented in Figs 4(d) and 5(e). Nano-size inclusions (~10–50 nm, white) were found inside these grains and high strain fields were observed around them. EDS analysis was applied on these distinct inclusions and several randomly selected spots in the matrix; those for spot C (matrix) and spot D (inclusion) are presented in Fig. 4(f). These EDS results confirm that these white inclusions were NbB_2_. A STEM-EDS line scan was applied across inclusion D. The intensity of Nb signals abruptly decreased from ~10^4^ to 0 across the NbB_2_/MgB_2_ interface and no Nb was detected in other regions of the MgB_2_ grains.

The microstructure of HTP-DY (HT above the peritectic) was investigated by BSE in Fig. 5(a). Five phases are visible: MgB_2_ (majority phase, dark grey), Mg (main phase, grey), MgB_4_ (minority phase, black), MgO (minority phase, light grey) and Dy-containing inclusions (minority phase, white). These Dy- containing inclusions (DyB_4_ according to XRD results) with a size of ~100 nm were dispersed throughout the bulk. Bright-field TEM examination revealed a number of impurity phases in the form of ~10–50 nm inclusions inside the MgB_2_ grains in Fig. 5(b). A low density of large inclusions (over 100 nm) was also observed (Fig. 5(c)). HAADF imaging (Fig. 5(c)) showed that these nano-size inclusions had a higher average atomic weight. EDS indicates that these inclusions contained Dy and B suggesting they are the previously XRD-identified DyB_4_. STEM-EDS analysis was applied across inclusion B (the red dashed line in Fig. 5(c)) and the result is shown in Fig. 5(e). No Dy was detected outside the inclusion.

### Influence of MB_2_ and Dy_2_O_3_ doping on superconducting properties - Magnetic Results

**Figure 6:**
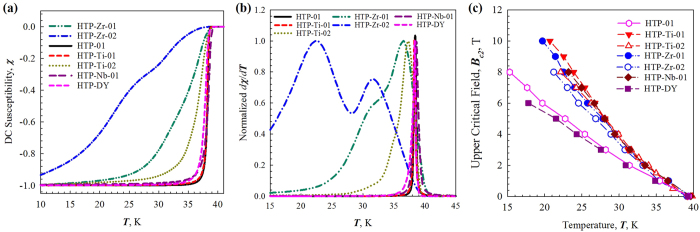
(**a**) DC susceptibility χ vs *T* at 0.01 T; (**b**) the *T*_*c*_ distribution - dχ/d*T* vs *T*; and (**c**) the temperature dependent *B*_*c2*_ (*T*) curves of the all MgB_2_ samples.

The superconducting transition temperature *T*_*c*_ and the distribution of *T*_*c*_ of all samples were extracted by magnetization measurements, Fig. 6. The onset *T*_*c*_s and the full-width half maximum (FWHM) of all samples are listed in Table 2. The undoped sample HTP-01 shows a very sharp superconducting transition with *T*_*c*_ of 39.5 K and a FWHM of ~0.4 K. Below we describe the results for the doped samples.

### ZrB_2_ Doping

In the ZrB_2_ doped samples, the onset *T*_*c*_s is ~39.2 K in HTP-Zr-01 and ~39.4 K in HTP-Zr-02, respectively. The unchanged *T*_*c*_s suggest that a portion of these ZrB_2_ doped samples was unaffected with a *T*_*c*_ equal to that of the undoped sample. Figures 6(a,b), show very broad transitions and bi-modal peak in the *T*_*c*_ distribution. This effect became more severe in HTP-Zr-02 indicating the presence of regions with various *T*_*c*_s.

### TiB_2_ Doping

Similarly, in the TiB_2_ doped samples, the onset *T*_*c*_s were unchanged, while their FWHMs were increased to ~0.5 K in HTP-Ti-01 and ~2.8 K in HTP-Ti-02, respectively.

### NbB_2_ Doping

The NbB_2_ doped sample HTP-Nb-01 has an onset *T*_*c*_ of 39.8 K and a FWHM of ~1.0 K.

### Dy_2_O_3_ Doping

The Dy_2_O_3_ doped sample HTP-DY has an onset *T*_*c*_ of 39.2 K and a FWHM of ~0.5 K.

The results for each of the MB_2_ samples was similar-after MB_2_ doping, the onset *T*_*c*_s were relatively unaffected, however their transition widths were significantly enhanced. We interpret this effect in terms of the presence of nanoscale MB_2_ (where M = Zr, Nb, or Ti) second phases which produce locally distorted regions separated by large regions of unaffected MgB_2_, leading to broadened *T*_*c*_ distributions with a wide *T*_*c*_ variation ranging from 39 K to low values. Since these MB_2_ additives are isomorphous to MgB_2_ and their lattice parameters are close to those of MgB_2_, the localized distortion is probably due to the coherent strain generated around the MB_2_ inclusions. However, in HTP-DY both the onset *T*_*c*_ and FWHM did not change by adding Dy_2_O_3_, which is consistent with Chen’s observation^17^. The lattice parameters and crystal structures of Dy_2_O_3_ (cubic with space group Ia-3)^20^ and DyB_4_ (tetragonal with space group P4/mbm)^21^ are very different from MgB_2_ (hexagonal with space group P6/mmm)^22^, therefore it is unlikely that the Dy-contained inclusions in HTP-DY can generate coherent strain in the MgB_2_ grains.

As indicated above the MB_2_ dopants were mostly found as distinct impurity inclusions that only influenced the surrounding MgB_2_ grains through the MgB_2_/MB_2_ interfaces. Increasing the concentration of MB_2_ inclusions produced more “affected zones” leading to a wider *T*_*c*_ distribution, Fig. 6(b). The behaviors of MB_2_ doped samples are quite different from those of C-doped MgB_2_ bulks^6^. After doping with 6.2 at.% C Susner *et al*.^6^ observed a significant decrease in the onset *T*_*c*_ , from 39.5 K to ~24 K, while the FWHM changed from 0.65 K to 1.4 K^6^. Since C is known to be a substitutional defect, if homogeneous C doping is achieved, the onset *T*_*c*_ and the lattice parameter *a* will decrease simultaneously with increasing C doping levels^6^. Under MB_2_ doping, it seems that Zr, Ti and Nb did not substitute for Mg or form homogeneous solid solutions with MgB_2_, even under 1700 °C and 10 MPa. However, the properties of the host lattices in the vicinities of these dopants were indeed affected and their *T*_*c*_s were clearly altered, possibly due to local compositional changes caused either by Mg diffusion into MB_2_ particles or by local strain. Both of these possibilities could cause *T*_*c*_ reduction, comparable to the effect of Al doping in MgB_2_^23^,^24^,^25^. Based on the results in the previous section, the affected vicinities probably had thicknesses similar to or smaller than 5 nm-the resolution of STEM-EDS line scans used in this study.

The *T* dependencies of the upper critical fields *B*_*c2*_ of all the samples are presented in Fig. 6(c). The *B*_*c2*_s at 20 K linearly extrapolated from Fig. 6(c) are listed in Table 2. It is clear that *B*_*c2*_ was increased by MB_2_ doping, but not by Dy_2_O_3_ doping. It is important to note, however, that since these MB_2_ doped samples were not homogeneous (as evidenced by the microstructure and the *T*_*c*_ distribution), the *B*_*c2*_ values represent the properties of only a small fraction of the bulk samples. In other words, some “affected zones” inside these doped bulks have higher *B*_*c2*_s than those in the unaffected MgB_2_, therefore their measured *B*_*c2*_ was enhanced. The observed high defect densities in the MgB_2_ grains, which increase electron scattering and reduce the electron mean free path, are likely responsible for the *B*_*c2*_ enhancement. These regions are at the edge of the grains, and can therefore act as connected percolative paths. In the Dy_2_O_3_ doped sample, although nano-size inclusions were observed inside the MgB_2_ grains, *B*_*c2*_ did not change. This observation together with the absence of changes in the lattice parameters, and lack of change in the *T*_*c*_ and FWHM suggested that Dy_2_O_3_, unlike the metal diboride additions, did not cause a band of defect structure at the boundary of the MgB_2_ grain.

**Figure 7:**
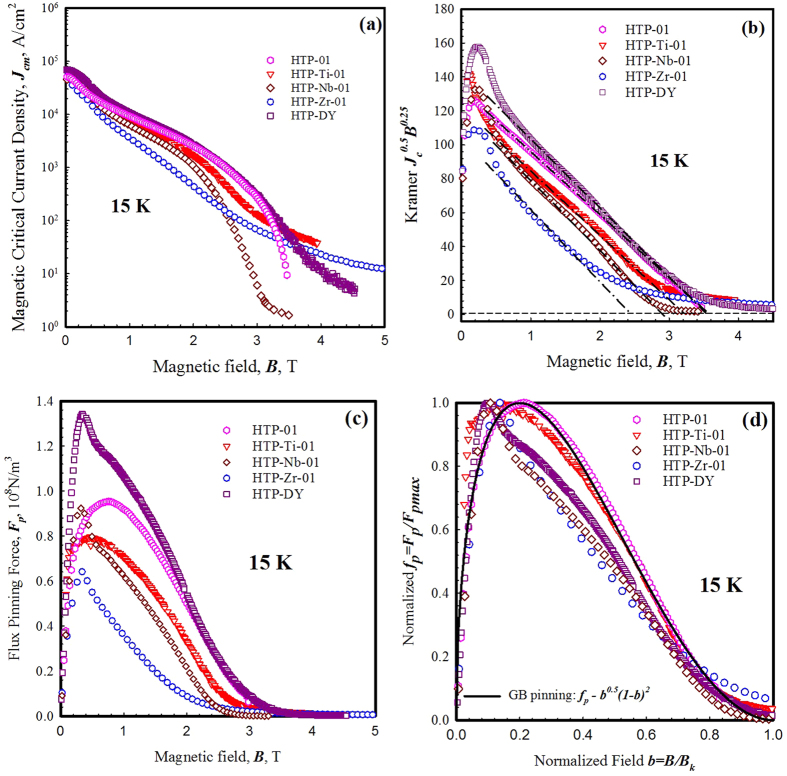
(**a**) Magnetic critical current density *J*_*cm*_ vs *T*; (**b**) Kramer plot *J*_*c*_^0.5^*B*^0.25^ vs *B*; (**c**) The flux pinning density *F*_*p*_ vs *B*; and (**d**) Normalized flux pinning behavior *f*_*p*_ vs *b* of the undoped sample HTP-01 and the doped samples HTP-Zr-01, HTP-Ti-01, HTP-Nb-01 and HTP-DY at 15 K.

The magnetic critical current density *J*_*cm*_s and flux pinning behaviors of selected samples were calculated based on Bean’s critical state model:


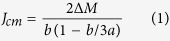


where Δ*M* is the width of the hysteresis loop at a given field *B, a* and *b* are the edge lengths of the sample orthogonal to B (*a* > *b*). The results at 15 K are shown in Fig. 7. The *J*_*cm*_s for most of the field range were either not changed, or even reduced after MB_2_ doping; for HTP-DY, its *J*_*cm*_ was slightly increased at all measured fields. A “tail” in *J*_*cm*_(*B*) can be observed in all MB_2_ doped samples, Fig. 7(a). Based on the microstructural evidence and results of *B*_*c2*_ and *T*_*c*_, this “tail” in *J*_*cm*_ of the MB_2_ doped samples is probably caused by regions in the samples with different *B*_*c2*_s. However these regions were too small to have significant influence on the overall *J*_*c*_ (>100 A/cm^2^). The irreversibility field, *B*_*irr*_, defined as the point where flux pinning vanishes, is often taken as the field at which *J*_*c*_ (*B*) = 100 A/cm^2^. The results for these samples are given in Table 2; no increase in *B*_*irr*_ is seen, and in some cases there is a decrease. This definition of *B*_*irr*_ does not capture the high field and super-low-*J*_*c*_ “tail” observed in Fig. 7(a).

MgB_2_ is primarily a grain boundary pinner, and thus the starting place to describe its pinning is the Kramer function (although deviations are seen). In order to perform such analysis, a Kramer field is needed. A Kramer plot, *J*_*c*_^0.5^*B*^0.25^ versus *B*, is shown in Fig. 7(b). The Kramer fields, *B*_*k*_, taken at the cross-intercepts of linear fittings (Black dash lines in Fig. 7(b)) are listed in Table 2. The values of *B*_*k*_, similarly to those of *B*_*irr*_, were stable or slightly reduced after MB_2_ doping, unlike the values of *B*_*c2*_ which increased with doping. This effect is due to several factors: (1) the higher *B*_*c2*_ region was apparently small, presumably restricted to the defected zones near the grain boundaries, these regions will not substantially influence the measured *B*_*k*_; (2) the *B*_*k*_ is also affected by the sample connectivity, possibly reduced with second phases present. For samples with Dy_2_O_3_ additions, *B*_*c2*_, *B*_*irr*_, and *B*_*k*_ were not affected.

Figure 7 (c) is a plot of bulk pinning force density (*F*_*p*_ = *J*_*cm*_ × *B*) vs *B*; the maximum values, *F*_*pmax*_, are listed in Table 2. Compared to the literature values, the *F*_*p,max*_ of these HTP processed bulks is quite small. Spark-plasma sintering^26^ gives a *J*_*c*_ at 2 T and 15 K of about 10^5^ A/cm^2^, while our values here are closer to 2 × 10^3^ A/cm^2^. The *F*_*p,max*_ of the undoped sample HTP-01 is only ~0.095 GN/m^3^ while according to Susner^6^ at 15 K the *F*_*p,max*_ of an undoped MgB_2_ wire was about 2 GN/m^3^. The main reason for the difference is that the MgB_2_ grains in these HTP bulks (>5 μm) are much larger than those in the traditional synthesized samples (typically 30–500 nm). Other factors could also contribute to this difference in *F*_*p,max*_, including some reduction of connectivity by small amounts of MgO. However, as *J*_*c*_ ∝ 1/grain size, and the grain size in our samples is roughly 50 times larger than the highest performing MgB_2_, the grain size effect is expected to be dominant. Among all samples, the highest value of *F*_*p,max*_ (~0.135 GN/m^3^) was observed in HTP-DY. The normalized bulk pinning force density 
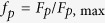
 is plotted against normalized magnetic field *b* = *B*/*B*_*k*_ in Fig. 7(d). The functions of grain boundary (GB) pinning from the Dew-Hughes model^27^, *f*_*p*_ ∝ *b*^*1/2*^(*1* − *b*)^2^, are also plotted for comparison. Although the undoped sample HTP-01 followed GB pinning function quite well, all doped samples show a deviation from the standard function. The peaks of *f*_*p*_ in the doped samples were shifted from the value of *b* = 0.2 (the peak position of the GB pinning) to lower values. For example, the peaks in HTP-Zr-01, HTP-Ti-01, HTP-Nb-01 and HTP-DY were 0.12, 0.13, 0.11 and 0.10, respectively. This observation which was also reported by Matsushita *et al*.^28^ in C doped MgB_2_ bulk samples can be explained by two possibilities: (1) These doped samples might contain a set of local *B*_*k*_s instead of one distinct value (just like the *B*_*c2*_s in the MB_2_ doped samples), which can lead to an artificial error in the estimation of the peak positions; while the variation in *B*_*k*_ of the undoped sample was small, thus the undoped sample followed the GB pinning function. (2) The deviation from *b*_*peak*_ = 0.2 might be caused by the operation of other pinning mechanisms (e.g., normal volume pinning in which *f*_*p*_ maximizes at *b* → 0.0 27) in association with the GB pinning. As noted above, we clearly see a distribution of Dy-based second phases, consistent with^17^. Also present were modest levels of MgO, known both to act as a pinner and in some cases reduce connectivity^29^. However, any small MgO effects should be present in all samples. The parameter *a* of the flux line lattice (FLL) is given by *a* = 1.07(*Φ*_*0*_/*B*)^1/2^, where *Φ*_*0*_ is the quantum of magnetic flux (*Φ*_*0*_ = 2.07 × 10^−15^ Wb). Based on this expression, the values of *a* vary from ~50 nm at 1 T to ~20 nm at 6 T. By definition, the size of the volume pins needs to be larger than *a*. Considering the fact that these doped samples contained intragrain inclusions some of which were bigger than the FLL parameter *a* at every measured field, it appears that volume pinning contributed to these shifts in *F*_*p,max*_.

In summary, after adding MB_2_, the *B*_*c2*_ of MgB_2_ HTP bulks increased, the *T*_*c*_ distributions were broadened, but *T*_*c*_, *B*_*k*_ and *J*_*c*_ remained unchanged (or slightly reduced). Considering the microstructural evidence, this observation can be explained as follows: only very small regions (possible ≤5 nm in thickness) around dopant particles of the MgB_2_ grains are influenced by doping, leaving the majority of MgB_2_ unaffected. To the contrary, the Dy_2_O_3_ doping did not change the *T*_*c*_, *T*_*c*_ distribution and *B*_*c2*_, instead it increased the *J*_*c*_ and flux pinning apparently associated with the nano-size precipitates in MgB_2_ grains.

## Conclusion

In this work we have used our HTP method for synthesizing doped MgB_2_ bulks at high temperatures (up to 1700 °C) and at pressure (10 MPa) to explore solubility limits of dopant species in MgB_2_, maximize diffusion, and (alternatively) attempt to form dense, nanoscale secondary phases during the sample synthesis. We explored both metal diborides (MB_2_, where M = Zr, Ti and Nb) for attempted Mg site substitution and Dy_2_O_3_ for nanoscale intragrain precipitate formation. Using the HTP process we conclusively show that the large increases in *B*_*c2*_ with metal diboride additions are due to a highly defected band within the grain, rather than substitution or inclusion within the grain, or grain boundary effects. High defect densities observed in MgB_2_ grains around/with these MB_2_ inclusions, cause electron scattering and therefore contribute to the *B*_*c2*_ enhancement and *T*_*c*_ distribution broadening. On the other hand, these regions (≤5 nm in thickness) were not large enough to significantly influence the high field *J*_*c*_, *B*_*irr*_, or *B*_*k*_. This observation explains the frequently observed increases seen for *B*_*c2*_ in materials with no accompanying increase in transport current. We also confirm the previously observed but sparsely distributed intragrain precipitates formed with Dy_2_O_3_ additions. Dy_2_O_3_ additions not change the lattice parameters, *T*_*c*_, *T*_*c*_ distribution and *B*_*c2*_ of MgB_2_, but increased the *J*_*c*_ and flux pinning by forming an array of nano-size precipitates in MgB_2_ grains.

## Methods

### Sample Synthesis

Three sets of MgB_2_ bulks with various MB_2_ (M = Zr, Ti and Nb) dopants were fabricated at high temperatures and under pressure. This HTP process^18^,^19^ based on the reactive liquid Mg infiltration (Mg-RLI) method^30^. Three metal borides with a structure isomorphous to MgB_2_ (P6/mmm) were selected as vectors for Mg-site substitution: ZrB_2_ (99.5%, Alfa Aesar), TiB_2_ (99.5%, Alfa Aesar) and NbB_2_ (99.5%, Alfa Aesar). As the Dy_2_O_3_ additive, Dy_2_O_3_ (>99.9%, <100 nm particle size, ALDRICH) was used. Amorphous B powder (50–100 nm in size) manufactured by Specialty Metals Inc.^31^,^32^ was hand mixed with the dopant powder and high energy ball milled for 15 min in an Ar atmosphere. These dopants and B powder mixtures were then pressed into ~8 mm tall by ~13 mm diameter pellets and placed in an MgO crucible. Mg turnings (~4 mesh, 99.98%, Alfa Aesar) were packed on top. The Mg:B ratio in the crucible was about 1:1 to avoid possible Mg deficiencies during heat treatment. This crucible was capped and placed inside the HTP autoclave (see also^18^,^19^). All samples were heat treated at 10 MPa in an Ar atmosphere. Two heat treatment routes were used: (1) heating up to 1500 °C and soaking for 30 min; (2) heating up to 1700 °C and soaking for 20 min. A slow cooling rate of 5 °C/min was used in both HT routes to maintain thermal equilibrium. The first route was designed to limit the temperature to just below the peritectic decomposition point of the reaction, thus preventing decomposition while maximizing the diffusion of the dopant species. The second route was designed to allow the reaction to occur on the temperature upswing, and hence to form MgB_2_ directly from MgB_4_ and Mg^+^ dopant species on cooling:


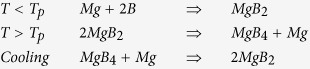


where *T*_*p*_ is the peritectic temperature (~1500 °C in our experiments).

### Measurements

A Rigaku SmartLab X-ray diffractometer (using Cu Kα of 1.5406 Å) was used for structural characterization and the scanning angle 2*θ* ranged from 20° to 80°. A FEI/Philips Sirion scanning electron microscope (SEM) with a field-emission source and a through-the-lens (TTL) detector was used for microstructural imaging. An FEI Helios 600 dual beam focused ion beam instrument (FIB) with an Omniprobe micromanipulation tool was used to prepare TEM thin foils. The TEM imaging was performed on a FEI/Philips CM-200T transmission electron microscope (TEM) with a silicon drift detector (SDD) and energy-dispersive X-ray spectroscopy function (EDS). The high-angle annular dark-field imaging (HAADF) and EDS line scans with a resolution of ~5 nm were performed on a Tecnai F20 system field emission 200 kV scanning transmission electron microscope (STEM) with an X-TWIN lens and high brightness field emission electron gun (FEG).

The magnetic properties were measured by using a Quantum Design Model 6000 PPMS with 4.2 K < *T* < 300 K and −10 T < *B* < 10 T. The superconducting critical transition temperature *T*_*c*_ and *T*_*c*_ distribution were determined by DC magnetic susceptibility methods. The *T*_*c*_ was defined as the onset of superconductivity from the normal state at 10 mT and the *T*_*c*_ distribution was expressed in terms of dχ/d*T*, where χ is the DC susceptibility. *M-T* curves were taken at 1 T intervals from 0–14 T. The upper critical field, *B*_*c2*_, was determined by the highest temperature point where the *M-T* curves deviated from *M* = 0 at each given field. The irreversibility field, *B*_*irr*_, was taken as the field at which *J*_*c*_(*B*) = 100 A/cm^2^. The Kramer field, *B*_*k*_, was taken as the point where *J*_*c*_^*0.5*^*B*^*0.25*^ extrapolated to zero on a Kramer plot (Fig. 7(b)), and the bulk pinning force density, *F*_*p*_, was calculated from *F*_*p*_ = *J*_*c*_*B*, where *J*_*c*_ was extracted from the magnetization results at various temperatures.

## Additional Information

**How to cite this article**: Yang, Y. *et al*. Influence of Metal Diboride and Dy_2_O_3_ Additions on Microstructure and Properties of MgB_2_ Fabricated at High Temperatures and under Pressure. *Sci. Rep.* **6**, 29306; doi: 10.1038/srep29306 (2016).

## Figures and Tables

**Table 1:** Doping levels, heat-treatment parameters, lattice parameters and MB_x_ impurity amounts for the MgB_2_ samples.

Sample	Dopant:B ratios^1^	HTs at 10 MPa	*a*, Å	*a*_cal_, Å^2^	*c* Å	*c*_cal_, Å	MB_x_ Impurities^3^
HTP-01	—	1500 °C, 30 min	3.082(5)	—	3.521(7)	—	—
HTP-Zr-01	ZrB_2_:B = 1:40	1500 °C, 30 min	3.086(4)	3.085	3.530(1)	3.522	1 wt% ZrB_2_
HTP-Zr-02	ZrB_2_:B = 1:19	1700 °C, 20 min	3.090(2)	3.091	3.527(4)	3.522	8.6 wt% ZrB_2_
HTP-Ti-01	TiB_2_:B = 1:40	1500 °C, 30 min	3.089(3)	3.082	3.523(8)	3.516	1.7 wt% TiB_2_
HTP-Ti-02	TiB_2_:B = 1:19	1700 °C, 20 min	3.085(4)	3.077	3.520(2)	3.492	11.3 wt% TiB_2_
HTP-Nb-01	NbB_2_:B = 1:40	1500 °C, 30 min	3.086(3)	3.084	3.517(4)	3.517	2.7 wt% NbB_2_
HTP-Dy	Dy_2_O_3_:B = 1:800	1700 °C, 20 min	3.082(9)	—	3.522(5)	—	1.4 wt% DyB_4_

^1^These ratios were based on the mole ratios between the dopants and B in the mixtures.

^2^The *a*_*cal*_ and *c*_*cal*_ were the calculated values based on Vegard’s law by assuming that all dopants form a homogeneous solid solution with MgB_2_.

^3^The x in MB_x_ are 2 in the MB_2_ doped samples and 4 in the Dy_2_O_3_ doped sample, respectively.

**Table 2:** Comparison of the superconducting properties amount the MgB_2_ samples.

Sample	Onset *T*_*c*_ (K)	FWHM of *T*_*c*_(K)	*B*_*c2*_ at 20 K (T)	*B*_*k*_ at 15 K (T)	*B*_*irr*_ at 15 K (T)	*F*_*pmax*_(GN/m^3^)
HTP-01	39.5	0.4	5.8	3.58	3.24	0.095
HTP-Zr-01	39.2	~10	10	2.47	2.66	0.063
HTP-Zr-02	39.4	>15	8	—	—	—
HTP-Ti-01	39.5	0.5	10	3.27	3.15	0.079
HTP-Ti-02	39.6	2.8	8.2	—	—	—
HTP-Nb-01	39.8	1.0	9	2.88	2.56	0.091
HTP-DY	39.2	0.5	5.6	3.51	3.30	0.134
